# Transdermal Estrogen in Women With Anorexia Nervosa: An Exploratory Pilot Study

**DOI:** 10.1002/jbm4.10251

**Published:** 2019-12-10

**Authors:** Megi Resulaj, Sai Polineni, Erinne Meenaghan, Kamryn Eddy, Hang Lee, Pouneh K Fazeli

**Affiliations:** ^1^ Neuroendocrine Unit Massachusetts General Hospital Boston MA USA; ^2^ Department of Psychiatry Massachusetts General Hospital Boston MA USA; ^3^ Biostatistics Center Massachusetts General Hospital Boston MA USA; ^4^ Harvard Medical School Boston MA USA; ^5^ University of Pittsburgh School of Medicine Pittsburgh PA USA

**Keywords:** ANOREXIA NERVOSA, BONE MINERAL DENSITY, MARROW ADIPOSE TISSUE, TRANSDERMAL ESTROGEN

## Abstract

Anorexia nervosa (AN) is a psychiatric disorder characterized by self‐induced starvation, low body weight, and elevated levels of bone marrow adipose tissue (BMAT). BMAT is negatively associated with BMD in AN and more than 85% of women with AN have low bone mass and an increased risk of fracture. Although a majority of women with AN are amenorrheic, which is associated with low BMD, oral contraceptive pills, containing supraphysiologic doses of estrogen, are not effective in increasing bone mass. We performed a 6‐month, open‐label study of transdermal estradiol (0.045 mg/day) + levonorgestrel (0.015 mg/day) in 11 women with AN (mean age ± SEM: 37.2 ± 2.3 years) to investigate the effects of transdermal, physiologic doses of estrogen on BMD and BMAT in women with AN. We measured change in BMD by DXA, change in BMAT at the spine/hip by ^1^H‐magnetic resonance spectroscopy, and change in C‐terminal collagen cross‐links (CTX), P1NP, osteocalcin, IGF‐1, and sclerostin after 3 and 6 months of transdermal estrogen. Lumbar spine (2.0% ± 0.8%; *p* = 0.033) and lateral spine (3.2% ± 1.1%; *p* = 0.015) BMD increased after 6 months of transdermal estrogen. Lumbar spine BMAT decreased significantly after 3 months (−13.9 ± 6.0%; *p* = 0.046). Increases in lateral spine BMD were associated with decreases in CTX (*p* = 0.047). In conclusion, short‐term treatment with transdermal, physiologic estrogen increases spine BMD in women with AN. Future studies are needed to assess the long‐term efficacy of this treatment. © 2019 The Authors. *JBMR Plus* published by Wiley Periodicals, Inc. on behalf of American Society for Bone and Mineral Research.

## Introduction

Anorexia nervosa, a psychiatric disorder predominantly affecting women, is characterized by an inappropriately low body weight caused by self‐induced starvation. Over 85% of women with this disorder have low bone mass,^1^ which is associated with a significantly increased risk of fracture.^2^, ^3^ Approximately 30% of women with anorexia nervosa report a prior history of fracture,^1^ and a prospective study found a sevenfold increased risk of fracture in women with anorexia nervosa compared with normal‐weight women of similar age.^4^

Functional hypothalamic amenorrhea, a common finding in women with anorexia nervosa, is an adaptive response to chronic starvation, resulting in decreased energy expenditure on the costly process of reproduction during periods of nutritional insufficiency. Duration of amenorrhea is associated with low BMD in women with anorexia nervosa^5^; therefore, hypoestrogenemia is an important potential determinant of low bone mass in this population. Yet despite retrospective studies suggesting a potential beneficial effect of oral estrogen on bone in anorexia nervosa,^5^ prospective and randomized, placebo‐controlled studies have shown that oral estrogen, predominantly in the form of oral contraceptive pills that contain supraphysiologic doses of estrogen, does not increase BMD in women with anorexia nervosa.^6^, ^7^, ^8^, ^9^

In contrast, treatment with physiologic, transdermal estrogen for 18 months in adolescents with anorexia nervosa significantly increases BMD,^10^ but whether this will be effective in adult women with anorexia nervosa is not known. Prior treatment studies have shown substantial differences in response to treatments in adolescents with anorexia nervosa compared with adults. For example, compared with placebo, 1 year of bisphosphonate therapy increased spine and hip BMD in adults with anorexia nervosa by 2% to 4%,^11^ but did not show benefit in adolescents.^12^ This may be because of differences in bone modeling/remodeling in adolescents compared with adults with anorexia nervosa. Adolescent girls with anorexia nervosa have lower levels of bone formation markers and similar levels of bone resorption markers compared with normal‐weight adolescents,^13^, ^14^, ^15^ suggestive of decreased modeling/remodeling. In contrast, adults with anorexia nervosa have similarly suppressed levels of bone formation markers but increased levels of bone resorption markers compared with normal‐weight women,^16^, ^17^ suggesting increased bone resorption in adult women with anorexia nervosa compared with adolescents. Therefore, treatments that are effective in one of these groups may not be effective in the other.

An additional potential determinant of low bone mass in anorexia nervosa is bone marrow adipose tissue (BMAT). In contrast to subcutaneous and visceral fat, levels of BMAT are elevated in anorexia nervosa and inversely associated with BMD.^18^, ^19^ BMAT has been associated with parameters of impaired bone integrity, including vertebral compression fractures; therefore, it may contribute to increased fracture risk.^20^ To date, there are no studies investigating the effects of a treatment for low bone mass on BMAT in adult women with anorexia nervosa. Therefore, we performed an exploratory study investigating the effects of transdermal, physiologic estrogen replacement on BMD in adult women with anorexia nervosa. In addition to BMD, we also measured BMAT and hormones associated with mineral metabolism at 3 months and 6 months after initiation of transdermal estrogen to explore potential mechanisms underlying changes in BMD. We hypothesized that treatment with transdermal, physiologic estrogen replacement would increase BMD and decrease levels of BMAT in women with anorexia nervosa.

## Materials and Methods

### Subjects

We studied 11 premenopausal women with anorexia nervosa (mean ± SEM: 37.2 ± 2.3 years). Participants were recruited through online advertisements and referrals from local eating disorder providers. Subjects were interviewed by the team psychologist (KE) to ensure that they met DSM‐5^21^ criteria for anorexia nervosa. All of the subjects were amenorrheic and none had used exogenous estrogen within at least 3 months of their baseline visit. All subjects had a 25‐OH vitamin D level >20 ng/mL at the time of their screening visit. Subjects with abnormal thyroid function tests or chronic diseases known to affect BMD other than anorexia nervosa were excluded from participation. Participants were all being followed by an outpatient treatment team, including a primary care provider and/or therapist at baseline and throughout the duration of the study. No subject self‐reported a significant change in activity level during the course of the study, except for one subject who reported that she stopped running (she reported running 3 to 5 miles two or three times per week at baseline).

### Study protocol

All subjects were seen at the Translational and Clinical Research Center at the Massachusetts General Hospital (Boston, MA, USA) for a baseline visit, 3‐month study visit, and final (6‐month) study visit. After the baseline visit, subjects were initiated on a weekly transdermal patch (Climara Pro; Bayer Pharmaceuticals, Whippany, NJ, USA) containing both estradiol (0.045 mg/day) and the progestin levonorgestrel (0.015 mg/day). Although doses of transdermal estradiol as low as 0.014 mg/day have been shown to be effective for the treatment of low BMD in postmenopausal women,^22^ there are data suggesting there may be dose‐dependent effects up until about 0.075 mg/day.^23^ This product, which falls in the middle of this efficacy range, was chosen both because of the dose of estradiol administered and because it is the only hormone replacement therapy transdermal patch that also contains progesterone. Therefore, a single patch could be applied once weekly without the need for an additional progesterone product in women with an intact uterus, in whom unopposed estrogen may be contraindicated. Subjects replaced the patch weekly for 6 months. Subjects were also provided with supplements to achieve a daily intake of 1200 mg of calcium, and all subjects were provided with a daily vitamin containing 400 IU of vitamin D, although at baseline all but two subjects were ingesting at least 900 mg of elemental calcium daily through supplements and/or diet [mean daily intake of calcium through diet/supplements of all subjects (*n* = 11): 1300 mg/day ±188 mg/day (SEM)]. At each study visit, blood was drawn for laboratory studies, radiologic imaging (described below) was performed, and subjects were weighed on an electronic scale while wearing a hospital gown. Height was measured as the average of three readings on a single stadiometer at their first study visit. Frame‐size estimation was performed by caliper measurement of elbow breadth and compared with norms based on US National Health and Nutrition Examination Survey I data; percent ideal body weight was calculated based on 1983 Metropolitan Life Height and Weight tables.^24^ One subject stopped participation after 2 months in the study because of an inability to schedule follow‐up study visits. Two additional subjects completed the 3‐month study visit but discontinued participation thereafter: one subject discontinued participation because of scheduling difficulties and the development of breast tenderness/breast tissue growth, and the second subject discontinued participation because of exacerbation of symptoms associated with anorexia nervosa (increased lightheadedness).

The study was approved by the Partners HealthCare Institutional Review Board and complied with the Health Insurance Portability and Accountability Act guidelines. Written informed consent was obtained from all subjects.

### Radiologic imaging

#### *Dual energy X‐ray absorptiometry*

Subjects underwent DXA to measure areal BMD of the posterior–anterior lumbar spine (L1–L4), lateral spine (L2–L4), left total hip, and left femoral neck using a Hologic Discovery A densitometer (Hologic Inc., Bedford, MA, USA) at their baseline and final study visits. At the 3‐month study visit, areal BMD of the lumbar and lateral spine was also measured by DXA. Coefficients of variation of DXA have been reported as ≤1.08% for lumbar spine BMD and <2.2% for femoral neck BMD.^25^ Ten subjects completed the baseline and 3‐month study visit scans. Eight subjects completed the scans at all three study visits.

#### *^1^H‐magnetic resonance spectroscopy (^1^H‐MRS)*

^1^H‐magnetic resonance spectroscopy (^1^H‐MRS) was performed by the Metabolic Imaging Core of the Nutrition and Obesity Research Center at Harvard (Boston, MA, USA), as previously described.^18^

In brief, ^1^H‐MRS of the L4 vertebral body, the proximal femoral epiphysis, metaphysis, and mid‐diaphysis was performed to determine lipid content (Siemens Trio, 3 T; Siemens Medical Systems, Erlangen, Germany). For the L4 vertebra, a voxel measuring 15 × 15 × 15 mm (3.4 mL) was placed within the L4 vertebral body. Single‐voxel ^1^H‐MRS data were acquired using point‐resolved spatially localized spectroscopy (PRESS) pulse sequence without water suppression with the following parameters: TE of 30 ms, TR of 3000 ms, 8 acquisitions, 1024 data points, and receiver bandwidth of 2000 Hz. For the femur, a voxel measuring 12 × 12 × 12 mm (1.7 mL) was positioned within the proximal femoral epiphysis, and single voxel ^1^H‐MRS using the same non‐water‐suppressed PRESS pulse sequence was performed. This process was repeated with voxel placement in the metaphysis at the intertrochanteric region and the mid‐diaphysis. Automated procedures for optimization of gradient shimming and transmit and receive gain were used. The coefficient of variation (CV) for marrow fat quantification in this population at the reported sites, determined by scanning five subjects twice, has been reported as 3%.^26^

Fitting of the ^1^H‐MRS data was performed using LCModel software (version 6.1‐4A; Stephen Provencher, Oakville, ON, Canada). Data were transferred from the scanner to a Linux workstation, and metabolite quantification was performed using eddy current correction and water scaling. A customized fitting algorithm for bone marrow analysis provided estimates for all lipid signals combined (0.9, 1.3, and 2.3 ppm). LCModel bone marrow lipid estimates were automatically scaled to unsuppressed water peak (4.7 ppm) and expressed as lipid/water ratio.

### Biochemical assessment

IGF‐1 was measured by a luminescent immunoassay analyzer (ISYS Analyzer; Immunodiagnostics Corp., Woburn, MA, USA). The detection limit for IGF‐1 was 4.4 ng/mL, with an intra‐assay CV of 2.2% and an interassay CV of 5.1%. N‐terminal propeptide of type 1 procollagen (P1NP) and osteocalcin, both markers of bone formation, and C‐terminal collagen cross‐links (CTX), a marker of bone resorption, were also measured by a luminescent immunoassay analyzer (ISYS Analyzer; Immunodiagnostics Corp.). For P1NP, the intra‐assay CV was 3.0% and the interassay CV was 5.0%. For osteocalcin the intra‐assay CV was 3.0% and the interassay CV was 6.0%. For CTX, the intra‐assay CV was 3.2% and the interassay CV was 6.2%. Sclerostin was measured by ELISA (R&D Systems, Minneapolis, MN, USA) with an intra‐assay CV of 2.0% and an interassay CV of 9.5%.

### Statistical analysis

Statistical analysis was performed using JMP Pro 13.0 (SAS Institute, Cary, NC, USA) software. If the data were normally distributed, means and standard error of the mean (SEM) were reported and compared using the Student's *t* test. If the data were not normally distributed, medians and the interquartile range were reported and compared using the Wilcoxon test. Paired sample *t* tests or Wilcoxon signed rank test (if data were nonnormally distributed) were used to compare changes in BMD and BMAT parameters between the study visits. To develop new hypotheses, we assessed univariate associations between changes in biologically plausible hormonal parameters and changes in BMD and BMAT in response to transdermal estrogen as part of this exploratory study; given the small sample size (*n* = 8 study completers), Spearman's coefficients were calculated to assess these univariate relationships. Repeated measures analysis was performed to investigate changes with time for CTX, P1NP, osteocalcin, and sclerostin using the baseline, 3‐month, and 6‐month timepoints. A *p* value of <0.05 was considered significant.

## Results

### Baseline characteristics of study population

Baseline characteristics of the study subjects are listed in Table 1. Subjects were a mean of 76.2% ± 2.1% of ideal body weight and had anorexia nervosa for a median (interquartile range [IQR]) of 16 [10, 23] years. Subjects participating in the study were amenorrheic for a median of 157 [36, 180] months and 27% (*n* = 3) of subjects reported a history of a stress fracture. Participants reporting a history of stress fracture had significantly lower BMD at the total hip and femoral neck as compared with participants reporting no prior history of a stress fracture (total hip BMD: history of stress fracture: median [IQR]: 0.601 g/cm^2^ [0.580 g/cm^2^, 0.689 g/cm^2^] versus no stress fracture history: 0.800 g/cm^2^ [0.719 g/cm^2^, 0.833 g/cm^2^], *p* = 0.032; femoral neck BMD: history of stress fracture: 0.528 g/cm^2^ [0.505 g/cm^2^, 0.611 g/cm^2^] versus no stress fracture history: 0.665 g/cm^2^ [0.638 g/cm^2^, 0.716 g/cm^2^], *p* = 0.032). Two additional participants, who did not have a history of a stress fracture, reported a history of a prior traumatic fracture; there were no significant differences in BMD at any site in individuals with a history of any fracture (*n* = 5) compared with those with no history of fracture (*p* = 0.315 to 0.927).

**Table 1:** Baseline Characteristics of the Study Participants

	Anorexia nervosa (*n* = 11)
Age (years)	37.2 ± 2.3
BMI (kg/m^2^)	17.8 ± 0.5
% of ideal body weight	76.2 ± 2.1
Number of years since onset of anorexia nervosa	16 [10, 23]
Duration of amenorrhea (months)	157 [36, 180]
History of stress fracture	27% (*n* = 3)
History of any prior fracture (stress or traumatic)	45% (*n* = 5)
% self‐reporting at least 10 hours/week of physical activity or running more than 10 miles per week	55% (*n* = 6)
25‐OH vitamin D (ng/mL)	38.2 ± 5.1
IGF‐I (ng/mL)	150 [140, 160]
P1NP (ng/mL)	49.5 [35.9, 71.1]
Osteocalcin (ng/mL)	11.7 [7.2, 31.4]
CTX (ng/mL)	0.51 ± 0.09
Sclerostin (pg/mL)	91.7 ± 4.8
BMD	
Lumbar spine (L1–L4) (g/cm^2^)	0.78 ± 0.03
Lumbar spine *T*‐score	−2.47 ± 0.28
Lumbar spine *Z*‐score	−2.24 ± 0.30
Lateral spine (L2–L4) (g/cm^2^)	0.60 ± 0.03
Lateral spine *T*‐score	−2.59 ± 0.33
Lateral spine *Z*‐score	−1.96 ± 0.30
Total hip (g/cm^2^)	0.74 ± 0.03
Total hip *T*‐score	−1.69 ± 0.24
Total hip *Z*‐score	−1.52 ± 0.23
Femoral neck (g/cm^2^)	0.64 ± 0.02
Femoral neck *T*‐score	−1.89 ± 0.20
Femoral neck *Z*‐score	−1.62 ± 0.20
Bone marrow adipose tissue	
L4 vertebra (lipid/water)	1.05 ± 0.12
Femoral metaphysis (lipid/water)	5.47 [3.44, 7.87]
Femoral diaphysis (lipid/water)	7.28 ± 0.73
Femoral epiphysis (lipid/water)	7.43 ± 0.79

We report (mean ± SEM) or median [interquartile range] when data were not normally distributed.

### Changes in bone mineral density after 6 months of physiologic, transdermal estrogen

**Figure 1:**
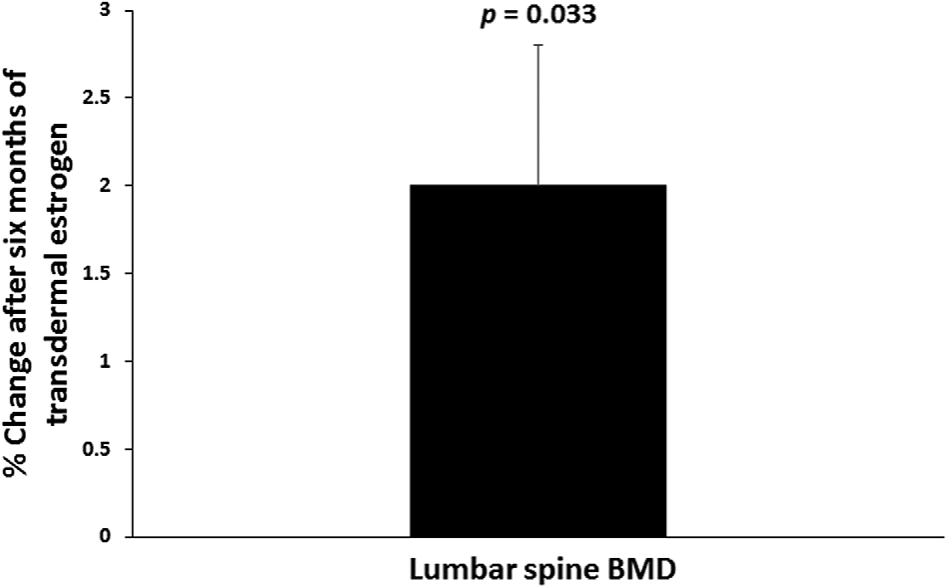
Lumbar spine BMD increased by 2.0% ± 0.8% (*p* = 0.033) after 6 months of treatment with transdermal estrogen in women with anorexia nervosa.

After 6 months of physiologic, transdermal estrogen, spine BMD increased significantly. Lumbar spine BMD increased by 2.0% ± 0.8% (*p* = 0.033) (Fig. 1 and Table 2), and lateral spine BMD increased by 3.2% ± 1.1% (*p* = 0.015). The mean change in weight for the group was 0.6% ± 2.3% over the 6‐month study. When we excluded the subject who gained more weight than the rest of the subjects (11.7% of initial body weight) and the subject who lost more weight than the rest of the subjects (−8.2% of initial body weight), the results remained significant (increase in lumbar spine BMD: 2.4% ± 0.9%, *p* = 0.033 and increase in lateral spine BMD: 3.6% ± 1.4%, *p* = 0.038). Total hip BMD (−0.6% ± 0.4%; *p* = 0.197) and femoral neck BMD (1.4% ± 1.0%; *p* = 0.181) did not change significantly over the 6‐month study.

**Table 2:** Bone Mineral Density, Bone Marrow Adipose Tissue, and Hormonal Parameters at Baseline and After 3 and 6 Months of Transdermal Estrogen

	Baseline(*n* = 11)	After 3 months of transdermal estrogen(*n* = 10)	After 6 months of transdermal estrogen(*n* = 8)	*p* value for 3 months versus baseline	*p* value for 6 months versus baseline
BMD					
Lumbar spine (g/cm^2^)	0.78 ± 0.03	0.79 ± 0.03	0.81 ± 0.04	0.072	**0.033**
Lateral spine (g/cm^2^)	0.60 ± 0.03	0.63 ± 0.03	0.64 ± 0.03	0.104	**0.015**
Total hip (g/cm^2^)	0.74 ± 0.03	Not measured	0.73 ± 0.04	**–**	0.197
Femoral neck (g/cm^2^)	0.64 ± 0.02	Not measured	0.65 ± 0.03	**–**	0.181
Bone marrow adipose tissue					
L4 vertebra (lipid/water)	1.05 ± 0.12	0.93 ± 0.11	0.98 ± 0.12	**0.046**	0.124
Femoral metaphysis (lipid/water)	5.47 [3.44, 7.87]	4.45 [2.35, 7.69]	4.28 [3.74, 6.04]	0.131	0.383
Femoral diaphysis (lipid/water)	7.28 ± 0.73	7.42 ± 1.10	7.71 ± 0.86	0.843	0.657
Femoral epiphysis (lipid/water)	7.43 ± 0.79	6.85 ± 0.97	7.80 ± 0.75	0.365	0.722
Hormonal parameters					
IGF‐1 (ng/mL)	150 [140, 160]	147 [138, 159]	149 [133, 174]	0.557	0.813
P1NP (ng/mL)	49.5 [35.9, 71.1]	58.2 [36.2, 82.2]	56.0 [42.8, 60.5]	0.695	0.469
Osteocalcin (ng/mL)	11.7 [7.2, 31.4]	12.2 [8.1, 31.9]	13.8 [8.6, 32.8]	0.770	0.469
CTX (ng/mL)	0.51 ± 0.09	0.39 ± 0.07	0.38 ± 0.09	**0.003**	**0.005**
Sclerostin (pg/mL)	91.7 ± 4.8	71.5 ± 3.8	84.5 ± 10.3	**0.011**	0.985

Mean ± SEM or median [interquartile range] when data were not normally distributed. *p* values <0.05 are in boldface.

CTX = C‐terminal collagen cross‐links.

### Changes in bone marrow adipose tissue with physiologic, transdermal estrogen

**Figure 2:**
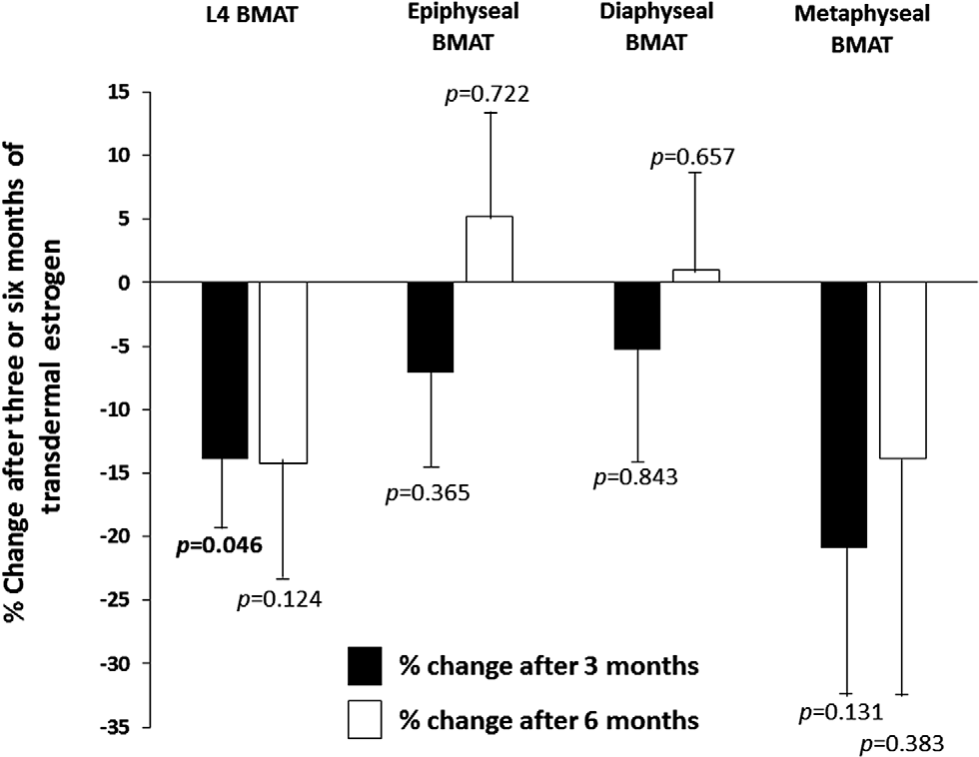
Bone marrow adipose tissue (BMAT) decreased significantly in the L4 vertebra after 3 months of treatment with transdermal estrogen in women with anorexia nervosa (*p* = 0.046). BMAT in the epiphysis, diaphysis, and metaphysis also decreased after 3 months of treatment, but the changes were not statistically significant. There were no significant changes in BMAT at the spine or hip after 6 months of treatment with transdermal estrogen. Three‐month changes are indicated by the black bars and 6‐month changes are indicated by the white bars.

After 3 months of physiologic, transdermal estrogen, BMAT at the spine decreased significantly. The change in L4 BMAT after 3 months was −13.9 ± 6.0% (*p* = 0.046) (Fig. 2). We have previously reported that subacute changes in weight are associated with changes in BMAT.^27^ Therefore, we performed a sensitivity analysis to ensure that changes in weight were not influencing our results. The mean 3‐month change in weight for the group as a whole was 0.3% ± 1.2%. When we excluded the participant who lost more weight (−8.4% of baseline weight) and the participant who gained more weight (4.9% of baseline weight) than the other study subjects after 3 months, the decrease in BMAT remained significant (change in L4 BMAT: −19.5% ± 6.2%, *p* = 0.023). The subject who gained more weight than the other study subjects was also the only subject to report a change in physical activity level during the course of the study (decrease in running); therefore, changes in physical activity level also did not affect the results. In the hip, after 3 months of treatment with transdermal estrogen, BMAT also decreased in the epiphysis (−7.1 ± 7.3%, *p* = 0.365), diaphysis (−5.3% ± 8.7%, *p* = 0.843), and metaphysis (−20.9% ± 11.5%, *p* = 0.131), but the changes were not statistically significant (Fig. 2). Similarly, after 6 months of treatment, there was a decrease in BMAT at the L4 vertebra compared with baseline (−14.2 ± 8.8%; *p* = 0.124), but the decrease did not achieve statistical significance. There were also nonsignificant changes in BMAT at the hip after 6 months of treatment with transdermal, physiologic estrogen (epiphysis: 5.2% ± 7.7%, *p* = 0.722; diaphysis: 1.0% ± 6.8%, *p* = 0.657; metaphysis: −13.9% ± 18.5%, *p* = 0.383) (Fig. 2). Changes in BMAT were not associated with changes in BMD during the 6‐month study.

### Changes in levels of markers of bone turnover, sclerostin, and IGF‐1

#### *CTX*

**Figure 3:**
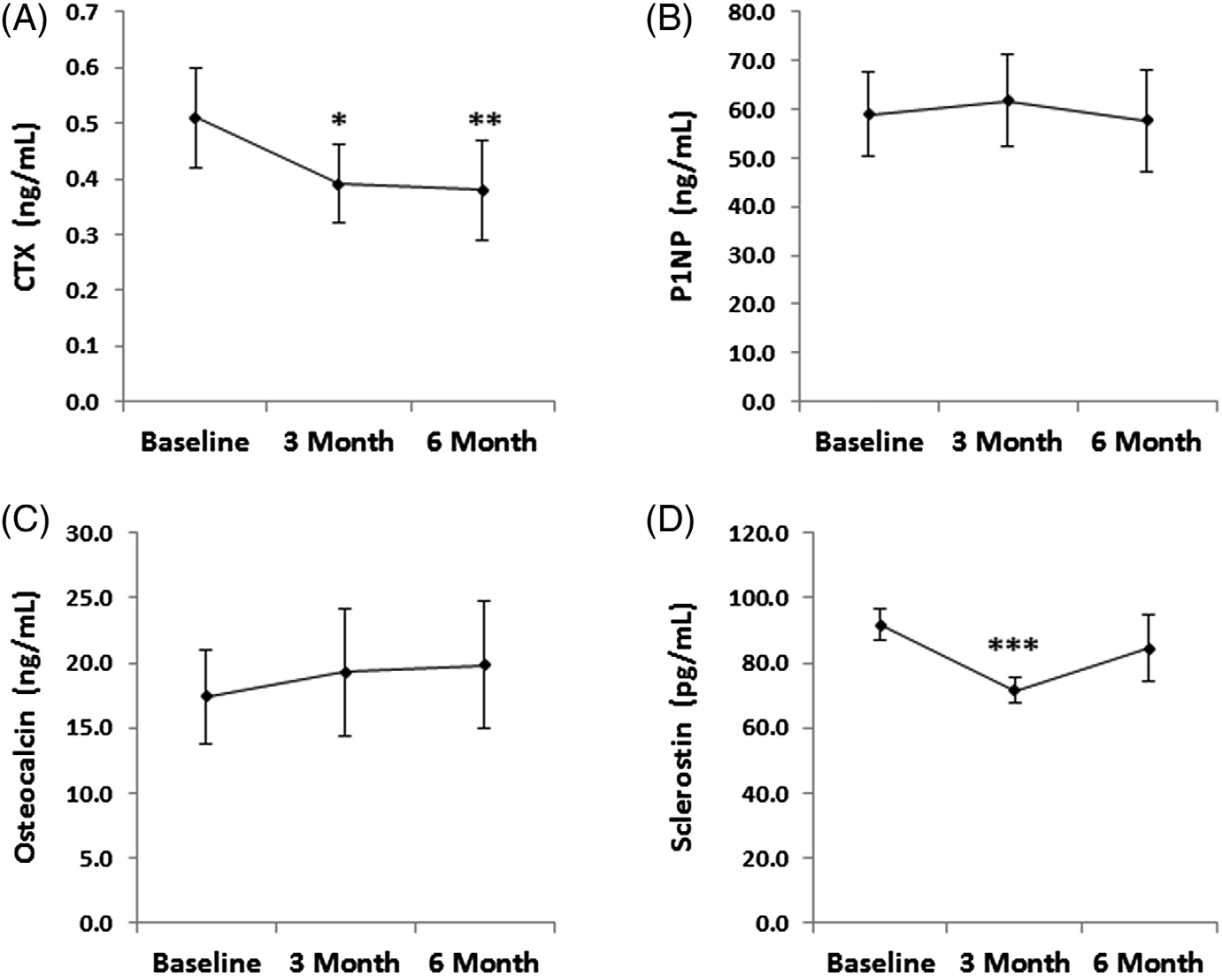
Changes in bone turnover markers with transdermal estrogen treatment. (*A*) C‐terminal collagen cross‐links (CTX), a marker of bone resorption, decreased significantly after 3 and 6 months of treatment with transdermal estradiol (repeated measures analysis, *p* = 0.028). (*B*,*C*) Levels of N‐terminal propeptide of type 1 procollagen (P1NP) (*B*) and osteocalcin (*C*), both markers of bone formation, did not change significantly with transdermal estradiol treatment (repeated measures analysis for P1NP, *p* = 0.457 and repeated measures analysis for osteocalcin, *p* = 0.313). (*D*) Sclerostin decreased significantly after 3 months of treatment with transdermal estradiol, but levels increased and were similar to baseline levels after 6 months of treatment. The overall pattern of change was not statistically significant with repeated measures analysis (*p* = 0.150). *p = 0.003 compared with baseline levels; **p = 0.005 compared with baseline; ***p = 0.011 compared with baseline (paired sample *t* test). Plotted values are Mean ± SEM.

Decreases in CTX, a marker of bone resorption, during the first 3 months of treatment with transdermal, physiologic estrogen predicted increases in lateral spine BMD over the 6‐month study period (rho = −0.71; *p* = 0.047) (Table 3). The mean % decrease in CTX after 3 months was −30.3% ± 6.0% and all study participants had lower CTX levels after both 3 months (*p* = 0.003) and 6 months (*p* = 0.005) of treatment with transdermal estrogen as compared with their baseline values (Fig. 3 *A*); compared with baseline levels, CTX levels decreased between −4.9% and − 63.9% after 3 months and between −21.7% and − 70.5% after 6 months of transdermal estrogen. The 6‐month change in CTX was predicted by the 3‐month change in diaphyseal BMAT (rho = −0.82; *p* = 0.023) (Table 3).

**Table 3:** Univariate Associations Between Changes in Biologically Plausible Hormonal Parameters and Changes in BMD and Bone Marrow Adipose Tissue in Response to Transdermal Estrogen (Spearman's Rho)

	3‐Month change in CTX	6‐Month change in CTX	3‐Month change in P1NP	6‐Month change in P1NP	3‐Month change in osteocalcin	6‐Month change in osteocalcin	3‐Month change in sclerostin	6‐Month change in sclerostin	3‐Month change in IGF‐1	6‐Month change in IGF‐1
3‐Month change in BMD										
Lumbar spine	rho = 0.25	Not assessed	rho = 0.27	Not assessed	= −0.01	Not assessed	rho = −0.44	Not assessed	**rho = 0.67**	Not assessed
	*p* = 0.489		*p* = 0.446		*p* = 0.987		*p* = 0.200		*p* **= 0.033**	
Lateral spine	rho = −0.21	Not assessed	rho = 0.30	Not assessed	rho = 0.24	Not assessed	**rho = −0.67**	Not assessed	rho = 0.44	Not assessed
	*p* = 0.556		*p* = 0.405		*p* = 0.511		*p* **= 0.033**		*p* = 0.200	
6‐Month change in BMD										
Lumbar spine	rho = −0.36	rho = −0.18	rho = 0.14	rho = 0.18	rho = 0.14	rho = −0.07	rho = 0.21	rho = −0.21	rho = −0.24	rho = 0.04
	*p* = 0.385	*p* = 0.702	*p* = 0.736	*p* = 0.702	*p* = 0.736	*p* = 0.879	*p* = 0.610	*p* = 0.645	*p* = 0.570	*p* = 0.939
Lateral spine	**rho = −0.71**	rho = 0.36	rho = 0.29	rho = 0.75	rho = −0.19	rho = 0.57	rho = −0.62	**rho = −0.82**	rho = 0.29	**rho = 0.79**
	*p* **= 0.047**	*p* = 0.432	*p* = 0.493	*p* =0.052	*p* = 0.651	*p* = 0.180	*p* = 0.102	*p* **= 0.023**	*p* = 0.493	*p* **= 0.036**
3‐Month change in bone marrow adiposity										
L4 vertebra	rho = −0.27	rho = −0.54	rho = 0.18	rho = −0.49	rho = −0.10	rho = −0.71	rho = −0.15	rho = −0.43	rho = 0.30	rho = −0.14
	*p* = 0.488	*p* = 0.266	*p* = .0.637	*p* = 0.329	*p* =0.798	*p* = 0.111	*p* = 0.700	*p* = 0.397	*p* = 0.433	*p* = 0.787
Femoral metaphysis	rho = −0.60	rho = −0.36	rho = 0.09	rho = 0.25	rho = 0.43	rho = −0.14	rho = −0.36	rho = −0.36	rho = 0.32	rho = −0.07
	*p* = 0.067	*p* = 0.432	*p* = 0.803	*p* = 0.589	*p* = 0.215	*p* =0.760	*p* = 0.310	*p* = 0.432	*p* = 0.366	*p* = 0.879
Femoral diaphysis	rho = −0.42	**rho = −0.82**	rho = 0.47	rho = −0.11	rho = 0.35	rho = −0.50	rho = −0.20	rho = −0.39	rho = 0.25	rho = −0.18
	*p* = 0.229	*p* **= 0.023**	*p* = 0.174	*p* = 0.819	*p* = 0.328	*p* = 0.253	*p* = 0.580	*p* = 0.383	*p* = 0.489	*p* = 0.702
Femoral epiphysis	rho = −0.30	rho = −0.77	rho = 0.17	rho = −0.54	rho = 0.18	**rho = −0.89**	rho = 0.13	rho = 0.20	rho = 0.13	rho = −0.77
	*p* = 0.433	*p* = 0.072	*p* = 0.668	*p* = 0.266	*p* = 0.637	*p* **= 0.019**	*p* = 0.732	*p* = 0.704	*p* = 0.732	*p* = 0.072
6‐month change in bone marrow adiposity										
L4 vertebra	rho = −0.50	rho = 0.37	rho = −0.04	rho = 0.09	rho = −0.71	rho = 0.03	rho = −0.68	rho = −0.43	rho = 0.71	rho = 0.37
	*p* = 0.253	*p* = 0.469	*p* = 0.939	*p* = 0.872	*p* = 0.071	*p* = 0.957	*p* = 0.094	*p* = 0.397	*p* = 0.071	*p* = 0.469
Femoral metaphysis	rho = −0.67	rho = −0.14	rho = −0.31	rho = 0.36	rho = 0.43	rho = 0.07	rho = −0.26	rho = −0.14	rho = −0.14	rho = −0.11
	*p* = 0.071	*p* = .760	*p* = 0.456	*p* = 0.432	*p* = 0.289	*p* = 0.879	*p* = 0.531	*p* = 0.760	*p* = 0.736	*p* = 0.819
Femoral diaphysis	rho = 0.14	rho = −0.43	rho = 0.36	rho = −0.11	rho = 0.02	rho = −0.21	rho = −0.05	rho = −0.46	rho = −0.50	rho = 0.14
	*p* = 0.736	*p* = 0.337	*p* = 0.385	*p* = 0.819	*p* = 0.955	*p* = 0.645	*p* = .0.911	*p* = 0.294	*p* = 0.207	*p* = 0.760
Femoral epiphysis	rho = −0.14	rho = −0.20	rho = −0.14	rho = −0.54	**rho = −0.79**	rho = −0.54	rho = 0.21	rho = −0.14	rho = −0.50	rho = −0.09
	*p* = 0.760	*p* = 0.704	*p* = 0.760	*p* = 0.266	*p* **= 0.036**	*p* = 0.266	*p* = 0.645	*p* = 0.787	*p* = 0.253	*p* = 0.872

*p* values <0.05 are in boldface.

CTX = C‐terminal collagen cross‐links.

#### *P1NP*

P1NP, a marker of bone formation, did not change significantly after either 3 months (*p* = 0.695) or 6 months (*p* = 0.469) of treatment with transdermal, physiologic estrogen (Fig. 3 *B*). There was a trend towards a significant positive association between change in P1NP over the 6‐month treatment period and change in lateral spine BMD after 6 months of treatment (rho = 0.75; *p* = 0.052) (Table 3). There were no associations between change in P1NP and change in BMAT (Table 3).

#### *Osteocalcin*

Osteocalcin, a marker of bone formation, did not change significantly after 3 months (*p* = 0.770) or 6 months (*p* = 0.469) of treatment with transdermal estrogen (Fig. 3 *C*). Change in osteocalcin after 6 months of treatment was predicted by 3‐month changes in epiphyseal BMAT (rho = −0.89; *p* = 0.019) (Table 3).

#### *Sclerostin*

Levels of sclerostin, a WNT inhibitor and negative regulator of bone formation, decreased significantly after 3 months of treatment with transdermal physiologic estrogen (−19.7% ± 5.6%; *p* = 0.011) (Fig. 3 *D* and Table 2). This result remained significant after excluding the one subject who reported a change in her physical activity level (decrease in running) during the study (−17.2% ± 5.7%; *p* = 0.022). The decrease in sclerostin after 3 months was significantly associated with the increases in lateral spine BMD observed after 3 months of treatment (rho = −0.67; *p* = 0.033) (Table 3). In contrast, levels of sclerostin after 6 months of treatment with transdermal, physiologic estrogen were not significantly different from baseline (1.4% ± 13.4%; *p* = 0.985), as levels of sclerostin increased between the 3‐month and 6‐month timepoints (Fig. 3 *D*). There were no associations between change in sclerostin and change in BMAT.

#### *IGF‐1*

**Figure 4:**
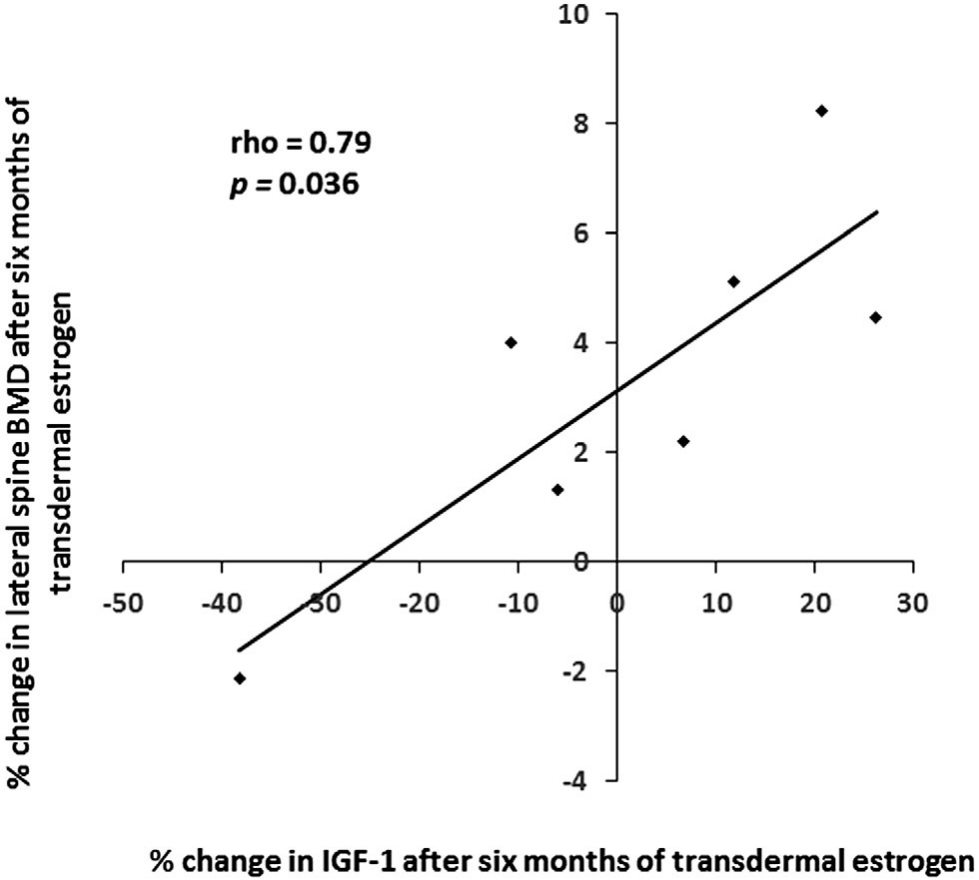
Change in levels of IGF‐1 after 6 months of treatment with transdermal estradiol was significantly associated with 6‐month change in lateral spine BMD in women with anorexia nervosa (rho = 0.79; *p* = 0.036).

IGF‐1 levels did not change significantly after 3 months (*p* = 0.557) or 6 months (*p* = 0.813) of treatment with transdermal estrogen (Table 2), but 3‐month changes in IGF‐1 were positively associated with 3‐month changes in lumbar spine BMD (rho = 0.67; *p* = 0.033) (Table 3). Similarly, 6‐month changes in IGF‐1 were significantly and positively associated with 6‐month changes in lateral spine BMD (rho = 0.79; *p* = 0.036) (Fig. 4 and Table 3). There were no associations between changes in IGF‐1 and changes in BMAT (Table 3).

### Adverse effects

The transdermal estradiol/levonorgestrel patch was well‐tolerated by study participants. Two subjects dropped out of the study after their 3‐month study visits. One subject, with a BMI of 16.4 at baseline and who reported being amenorrheic for the duration of her disease, stopped participation because of an exacerbation of lightheadedness (a symptom associated with the underlying disorder, anorexia nervosa). This subject had been on oral contraceptives in the past, but not for over 10 years prior to study participation. The second subject, who was 31 years old at the initiation of the study and reported 180 months of amenorrhea, discontinued participation because of difficulty scheduling visits and because she experienced breast tissue growth, as well as increased breast tenderness. This patient had also been on oral contraceptives for 24 months in the past, but not for 9 years prior to study participation. This patient reported being evaluated by her primary care physician and a breast surgeon, both of whom encouraged her to continue participation in the study, but the multiple visits required for evaluation of the breast tissue growth made it difficult to also continue with the scheduled study visits.

## Discussion

We have shown that short‐term treatment with transdermal, physiologic estrogen treatment increases spine BMD in adult women with anorexia nervosa. We have also found that vertebral BMAT, a possible determinant of low bone mass, decreases in response to estrogen replacement in this population. These data suggest that physiologic, transdermal estrogen replacement should be further studied as a possible treatment for low bone mass in adult women with anorexia nervosa.

Anorexia nervosa is a psychiatric disorder with a lifetime prevalence of approximately 2.2% in women,^28^ and the most common medical complication in women with anorexia nervosa is low bone mass. Annual rates of bone loss in adult, amenorrheic women with anorexia nervosa approach −2.6% in the spine and − 2.4% in the hip^29^. Although duration of amenorrhea is a strong predictor of low BMD,^5^ oral estrogen is not effective in increasing BMD as compared with placebo in this population.^6^, ^7^, ^8^, ^9^ In contrast, transdermal estrogen has been shown to be effective for the treatment of low bone mass in postmenopausal women^30^ and recently in adolescent girls with anorexia nervosa.^10^ Importantly, bone modeling and remodeling are distinctly different in adolescents with anorexia nervosa—who have an overall low rate of bone modeling with suppressed markers of bone formation^13^, ^14^, ^15^—and adults with anorexia nervosa—who have suppressed markers of bone formation, but increased levels of bone resorption.^(16,17)^ These contrasting modeling/remodeling rates may contribute to important differences observed in response to treatments for low bone mass in adults versus adolescents with anorexia nervosa; therefore, potential therapies must be independently studied in both populations.

Given the overall higher rate of bone resorption in adult women with anorexia nervosa as compared with adolescents, and the known effects of estrogen on bone turnover,^30^ we hypothesized that transdermal estrogen would also be an effective potential treatment for low bone mass in adult women with this disorder. We found a 2% increase in lumbar spine BMD after 6 months of treatment, which appears to be primarily caused by suppression of bone resorption, as decreases in CTX predicted the increase in lateral spine BMD. Although we did not observe changes in bone formation markers or IGF‐1, a bone anabolic hormone, with transdermal estrogen, we did observe significant positive association between changes in lateral and lumbar spine BMD and changes in IGF‐1. Levels of IGF‐1 are low in anorexia nervosa^31^, ^32^ and are associated with low bone mass^33^; oral estrogen's dose‐dependent suppression of IGF‐1^34^, ^35^ has been hypothesized to be an important contributor to the lack of benefit of oral estrogen for the treatment of low bone mass in anorexia nervosa.^10^ Our data suggest that in addition to suppression of bone resorption, IGF‐1 may also be a mediator of the beneficial effects of transdermal estrogen on BMD in this population. Therefore, whether longer‐term treatment with transdermal estrogen will be effective in increasing BMD in women with anorexia nervosa is an important question that warrants further study.

Sclerostin, an inhibitor of bone formation, has been associated with hip fractures in postmenopausal women,^36^ and a monoclonal antibody to sclerostin increases BMD^37^ and reduces fracture risk^38^ in this population. In contrast to adolescents with anorexia nervosa, in whom 12 months of treatment with transdermal estrogen did not result in significant changes in sclerostin levels,^39^ we found that 3 months of transdermal estrogen significantly lowered sclerostin levels, and that this decrease in sclerostin was associated with increases in lateral spine BMD. Importantly, after 3 months, sclerostin levels rose again in our study, suggesting that the effects of estrogen on sclerostin may only be short‐lived in anorexia nervosa and that sclerostin may be able to escape the effects of estrogen. Longer‐term studies will be necessary to better understand the potential effects of estrogen on sclerostin and its potential benefits to bone mass in women with anorexia nervosa.

Levels of BMAT, a component of the bone marrow microenvironment, are elevated in women with anorexia nervosa, despite low levels of subcutaneous and visceral adipose tissue.^18^ Although the function of BMAT is not known, it is inversely associated with BMD and hypothesized to be a determinant of low bone mass in anorexia nervosa.^18^ We found that 3 months of transdermal physiologic estrogen treatment significantly decreases vertebral BMAT, suggesting that estradiol may be an important determinant of BMAT in anorexia nervosa and consistent with data in postmenopausal women that show that 12 months of transdermal estradiol treatment decreases marrow adipocyte volume fraction.^40^ Whether these decreases in BMAT are associated with the observed increases in BMD remains unknown. BMAT has also been shown to be significantly associated with metabolic parameters including insulin resistance in women with anorexia nervosa^27^; therefore, BMAT may also have an important metabolic function. Interestingly, we found a significant association between change in BMAT and change in osteocalcin, a bone formation marker that has metabolic functions,^41^ including induction of both insulin and adiponectin, a hormone that has been shown to be secreted by BMAT in animal models.^42^ This association between osteocalcin and BMAT supports the concept that BMAT may serve a metabolic function and have a role in mineral metabolism as well.

Limitations of this exploratory pilot study include our small sample size and lack of a control group. Based on data from prior longitudinal studies, the annual rate of bone loss is estimated to be −2.6% in the spine for women with anorexia nervosa^29^; therefore, the finding of an increase in BMD in response to a treatment is one that warrants further study. As anorexia nervosa is a chronic disease from which only approximately 50% to 60% of women recover even two decades after diagnosis,^43^, ^44^ long‐term treatment options for low bone mass are critical. Unlike bisphosphonates or teriparatide, both of which improve BMD in adult women with anorexia nervosa,^11^, ^45^ but both of which also have limitations to long‐term use either because of the possibility of atypical femoral fractures (bisphosphonates)^46^ or FDA recommendations (teriparatide), there are no limitations to the long‐term use of transdermal estradiol. Therefore, longer‐term, randomized studies are necessary to study this potential treatment for low bone mass in women with anorexia nervosa.

## Disclosures

All authors state that they have no conflicts of interest.
